# Electrical Property Analysis of Textured Ferroelectric Polycrystalline Antimony Sulfoiodide Using Complex Impedance Spectroscopy

**DOI:** 10.3390/ma14102579

**Published:** 2021-05-15

**Authors:** Anna Starczewska, Bartłomiej Toroń, Piotr Szperlich, Marian Nowak

**Affiliations:** Institute of Physics–Center for Science and Education, Silesian University of Technology, Krasińskiego 8, 40-019 Katowice, Poland; anna.starczewska@polsl.pl (A.S.); piotr.szperlich@polsl.pl (P.S.); marian.nowak@polsl.pl (M.N.)

**Keywords:** antimony sulfoiodide (SbSI), ferroelectric, impedance spectroscopy, polycrystals, temperature dependences

## Abstract

Antimony sulfoiodide (SbSI) is a ferroelectric semiconductor with many interesting physical properties (optical, photoconductive, ferroelectric, piezoelectric, etc.). The electrical properties of textured polycrystalline SbSI obtained by the rapid cooling of a melted mass in liquid nitrogen are presented in this work using ac impedance spectroscopy over a wide temperature range (275–500 K) in the frequency range of 1 Hz to 100 kHz. Detailed studies of the impedance Z*(ω), conductivity σ*(ω), electric modulus M*(ω), and dielectric permittivity ε*(ω) of this material were performed using complex impedance spectroscopy for the first time. This study showed that the impedance and related parameters are strongly dependent on temperature. The internal domain structure and the presence of grain boundaries in textured polycrystalline SbSI explain the obtained results.

## 1. Introduction

Antimony sulfoiodide (SbSI) is a ferroelectric semiconductor with many interesting properties [1], including pyroelectric, pyro-optic, piezoelectric, electromechanical, and nonlinear optical effects. Crystalline SbSI has a chain structure and is one of the best piezoelectric crystals with a high volume piezoelectric modulus *d*_v_ = 1 × 10^−9^ C/N [2] and extremely high electromechanical coupling coefficient *k*_33_ = 0.90 [3]. The Pna2_1_ ($C_{2v}^{9}$) orthorhombic structure of the ferroelectric phase forms in SbSI crystals near room temperature. The phase transition temperature presumably depends on the growth method and chemical composition of the crystals [1,4,5] and is distributed from 283 K [6] to 298 K [7] for SbSI single crystals. A Curie temperature of 307 K was reported for SbSI polycrystals obtained by hot pressing [8]; however, the presence of impurities in SbSI electroceramics obtained by the Bridgeman method increases the Curie temperature (*T*_c_), even up to 331 K [9]. Doping SbSI single crystals with Cl atoms may also shift *T*_c_ up to 330 K [10]. In a paraelectric phase, i.e., above the phase transition temperature, the SbSI structure reforms into Pnma ($D_{2h}^{16}$), which was reported for SbSI crystals at 308 K [11,12,13] and 333 K [14]. It has also been reported that the SbSI crystal structure above room temperature is disordered, and its classification in any definite space group is only an approximation [15]. The authors of a previous study [15] suggested that from 298–410 K an orthorhombic space group of P2_1_2_1_2_1_ ($D_{2}^{4}$) is the best approximation. This indicates that SbSI becomes antiferroelectric with an alternating arrangement of polar double chains [(SbSI)_∞_]_2_ parallel to the [001] axis above the Curie point at 298 K; however, the same authors rectified this information based on a detailed study of diffuse scattering in the SbSI crystal structure at 320 K and reported that, in this case, the SbSI crystals exist as the paraelectric phase [16]. Indeed, the crystal structure in the paraelectric phase is described by the Pnma space group with displaced atoms. Each of the antimony, sulfur, and iodine atoms may occupy two positions, shifted up or down from the mirror plane perpendicular to the [001] direction by ΔSb = 30.6(14) pm, ΔS = 27(5) pm, and ΔI = 28.8(14) pm. The Reverse Monte Carlo (RMC) simulations of diffuse scattering confirmed the assumption that the [Sb(S,I)]_∞_ chains in the crystal structure of paraelectric SbSI at 320 K are not uniform but consist of sections with different lengths and opposite polarities. The distribution of these sections given by the RMC method reveals positively or negatively polarized irregular nanodomains that are several nm long, with transverse dimensions of about 0.5 nm × 2.0 nm and arranged along the [001] direction [16].

Recently, an antiferroelectric phase transition was reported for SbSI crystals grown by the Bridgman–Stockbarger technique, as shown by capacitance measurements at a frequency of 1 kHz [17]. The authors stated that SbSI has three phases: ferroelectric (*T* < 295 K), antiferroelectric (295 K < *T* < 410 K), and paraelectric (*T* > 410 K); however, the presence of a phase transition at *T* = 410 K was not confirmed by measurements performed on SbSI single crystals grown from the vapor phase [18]. These studies are very important for understanding the properties and possible applications of SbSI. For example, the aforementioned shifted positions of Sb, S, and I atoms may explain the recently reported piezoelectric properties of paraelectric phase SbSI-based nanocomposites [19,20,21]. 

The needle-like morphology was found to be predominant due to the specific inherent characteristics of the SbSI growth rate anisotropy; accordingly, it is difficult to grow good quality large single crystals or films [22,23,24]. The spontaneous polarization direction is parallel to the longitudinal *c*-axis of a crystalline needle; therefore, polarization characteristics are unmeasurable in untextured ceramics [25]. SbSI crystals tend to cleave parallel to the (110) plane, which makes them mechanically fragile; therefore, cutting thin plates perpendicularly to the cleavage axis and polishing any crystal surface is impossible [26,27,28]. An insufficient mechanical strength was reported even for textured polycrystalline SbSI grown by the Bridgman–Stockbarger technique [27,29,30,31]. Several methods have been proposed to overcome these disadvantages, e.g., impregnation of textured polycrystalline SbSI boules by low-viscosity epoxy resin under vacuum conditions [31,32].

Complex impedance spectroscopy (CIS) is a useful, non-destructive method for the analysis of material electrical properties over a wide range of frequencies and temperatures. It estimates the influence of bulk, grain boundary, and material electrode polarization on the electrical impedance. In this method, a single-frequency, sinusoidal, low-amplitude ac excitation signal is applied to a sample. The impedance magnitude |*Z*| and phase shift *φ* of the response current are measured. There are four fundamental functions for analyzing an impedance spectrum: impedance Z*(ω), conductivity σ^*^(ω), electric modulus M*(ω), and dielectric permittivity ε*(ω). The relations between them are as follows:

(1) $$Z^{*}(\omega )=\left|Z\right|\;(\omega {)e}^{i\varphi (\omega )}=Z{}^{\prime }\;+iZ{}^{\prime \prime }$$

(2) $$\sigma^{*}\left(\omega \right)=\frac{d}{A}\frac{1}{Z^{*}\left(\omega \right)}=\sigma {}^{\prime }+i\sigma {}^{\prime \prime }$$

(3) $$\varepsilon^{*}\left(\omega \right)=\frac{1}{C_{0}i\omega Z^{*}\left(\omega \right)}=\varepsilon {}^{\prime }\;-i\varepsilon {}^{\prime \prime }$$

(4) $$M^{*}\left(\omega \right)=\frac{1}{\varepsilon^{*}\left(\omega \right)}=M{}^{{}^{\prime }}+iM{}^{\prime \prime }$$

where *ω* = 2π*f* is the angular frequency, *d* is the sample thickness, *A* is a sample surface area, *C*_0_ = *ε*_0_*A*/*d* is geometric capacitance, *ε*_0_ is the permittivity of free space, and $i=\sqrt{-1}$.

A study of the ac electrical properties of solids indicates the various polarization mechanism and can also detect structural transitions and the charge transport mechanism in different regions or domains in the case of non-homogenous materials [33]. To the best of the authors’ knowledge and supported by an extensive literature survey, there has been no attempt to use complex impedance spectroscopy to understand the conduction mechanism in textured polycrystalline SbSI. There is growing interest in A^15^B^16^C^17^ compounds due to their physical properties, and SbSI is the main representative of these compounds. Therefore, this paper investigates the electrical properties of textured polycrystalline SbSI over a wide temperature range from 275 K to 500 K along the *c*-axis. 

## 2. Experiment

**Figure 1:**
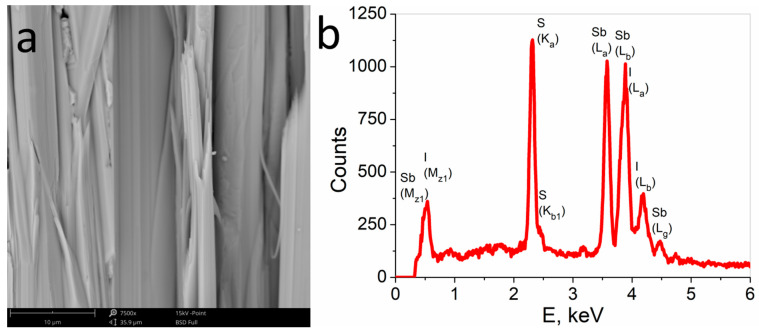
The SEM micrograph (**a**) and EDS spectrum (**b**) of textured polycrystalline SbSI.

Textured polycrystalline SbSI was prepared from a stoichiometric mixture of Sb_2_S_3_ and SbI_3_. The components were sealed in a closed evacuated Termisil glass ampoule at a pressure of 1 Pa. The ampoule was placed in a furnace for 24 h at 753 K to ensure the homogenous mixture of elements and was then slowly cooled to room temperature for 48 h. The obtained SbSI was crushed in a ceramic mortar and was rinsed in ethanol to remove unreacted SbI_3_ and other ethanol-soluble intermediates. The dry SbSI powder was sealed in a flat-bottomed Termisil glass ampoule evacuated to a pressure of 1 Pa. The SbSI was melted by heating it to 873 K (which is 200 K above the melting point of the material) and then it was rapidly cooled in liquid nitrogen. The textured polycrystalline SbSI has a diameter of 25 mm and a height of 8 mm. The morphology and chemical composition of the obtained material was examined by a Phenom PRO X (Thermo Fisher Scientific, Waltham, MA, USA) scanning electron microscope (SEM) equipped with an EDS analyzer. A typical SEM micrograph of the surface parallel to the temperature gradient during SbSI solidification is shown in Figure 1a, which clearly shows that the textured sample consisted of filament-like crystallites arranged along a single direction indicated by the temperature gradient. The EDS spectrum (Figure 1b) shows only peaks for antimony, sulfur, and iodine. The estimated elemental atomic ratio of the sample is 0.35:0.31:0.34 for Sb, S, and I, respectively, which confirms the good stoichiometry of textured polycrystalline SbSI. More details about sample preparation and material characterization are presented in [34].

A sample was cut from the synthesized material and ultrasonically cleaned to perform electrical investigations. Its surface area and thickness were 40.6 mm^2^ and 1.573 mm, respectively. SbSI crystallites grew along the temperature gradient during solidification, i.e., from the ampoule’s side surface to its center; therefore, the inclination angle α of the crystallites toward the sample surface can be calculated. The inclination angle α = 7.35° was estimated based on the ampule and sample dimensions since the sample was cut from the outer part of the solidified material. Crystallites inclined with a higher angle toward the surface plane were not observed in the SEM micrographs, but the local alignment may be slightly different due to crystallization along a local temperature gradient.

The largest opposite surfaces of the sample were covered with a silver paste (SPI Supplies, West Chester, PA, USA), and copper wires were attached to ensure an electrical connection. The sample was left for 48 h to evaporate the remaining solvent from the paste. Before measurement, the sample was kept at 500 K for 30 min to ensure electrode adherence and correct conduction between the electrodes and polycrystalline SbSI. The measurements were performed using an impedance analyzer (3533-50 LCR HITESTER, Hioki, Nagano, Japan) in the frequency range 1 Hz–100 kHz with a voltage amplitude of 100 mV and zero bias. The temperature from 275 K to 500 K was controlled by an R2205 Cryogenic Microminiature Refrigeration II-B System and K20 temperature controller (MMR Technologies, Inc., San Jose, CA, USA). The sample was kept at a constant temperature for 10 min before the measurement was performed to ensure it had reached thermal equilibrium. The measurements were performed in the dark to prevent excess photogeneration in the semiconductor. LabVIEW 2015 (National Instruments, Austin, TX, USA) was used for the computer-controlled experiments, data acquisition, and analysis. The Nyquist plots were fitted with an equivalent circuit using Complex Nonlinear Last Squares (CNLS) using the LEVM program developed by James Ross Macdonald (Chapel Hill, NC, USA) [35].

## 3. Results and Discussion

### 3.1. Dielectric Studies

**Figure 2:**
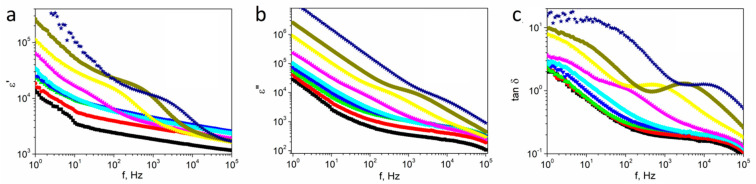
Real *ε*′ (**a**) and imaginary *ε*″ (**b**) part of the complex dielectric constant, and dielectric loss tangent (**c**) vs. frequency of textured polycrystalline SbSI at various temperatures ■ 280 K; ⬤ 290 K; ▲ 300 K; **▼** 310 K; ♦ 320 K; ◀ 350 K; ► 400 K; ⬢ 450 K; ★ 500 K.

The frequency spectra were recorded from 1 Hz to 100 kHz of the real (*ε*′) and imaginary (*ε*″) part of the complex dielectric constant (*ε* = *ε*′ − *iε*″) and dielectric loss tangent ($\tan\delta =\frac{\varepsilon {}^{\prime \prime }}{\varepsilon {}^{\prime }}$) for textured polycrystalline SbSI at selected temperatures between 275 K and 500 K (Figure 2).

The *ε*′ and *ε*″ values decreased upon increasing the frequency over the whole investigated temperature range. The plots show strong dispersion in the dielectric constant at low frequencies. In the high-frequency region, the values of *ε*′ and *ε*″ decreased to small values and became approximately independent of the frequency. Dielectric dispersion in some materials can be interpreted based on space-charge polarization. According to Maxwell and Wagner’s two-layer model [36], space-charge polarization arises due to the inhomogeneous dielectric structure of a material that is composed of large well-conducting grains separated by thin, low-conductivity intermediate grain boundaries. Furthermore, the nature of dielectric constant frequency changes is also dependent on temperature. The step-like relaxation behavior of the dielectric constant appears above 320 K and moves towards a higher frequency as the temperature increases. Besides, two different negative slopes of the log–log plot are observed in Figure 2b, and both moved to the high-frequency side as the temperature increased. This is a natural result of the frequency-independent conduction mechanism [37].

The variations of the same origin are also visible in the frequency spectrum of tan δ. A dielectric loss peak is observed at temperatures higher than 370 K in Figure 2c. Furthermore, the peak moved to higher frequencies upon increasing the temperature, and its value was between 1.25 and 1.3, reaching a maximum at 440 K.

**Figure 3:**
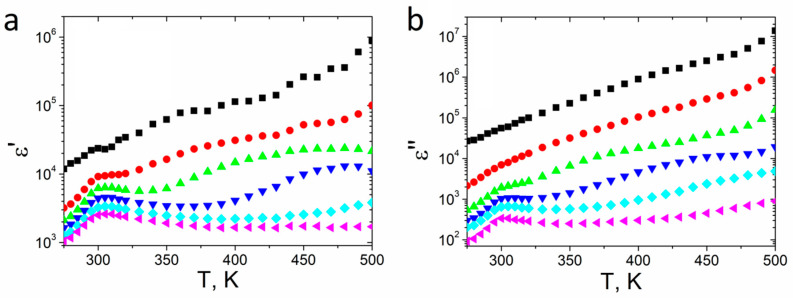
Real *ε*′ (**a**) and imaginary *ε*″ (**b**) part of complex dielectric constant vs. temperature of textured polycrystalline SbSI at selected frequencies (■ 1 Hz; ⬤ 10 Hz; ▲ 100 Hz; **▼** 1 kHz; ♦ 10 kHz; ◀ 100 kHz;).

The real (*ε*′) and imaginary (*ε*″) parts of the complex dielectric constant at selected frequencies as a function of temperature are shown in Figure 3. The values of the dielectric constant are high at low frequencies (*f* ≤ 100 Hz), and its value increased with the temperature. A dielectric peak at 300 K was observed at higher frequencies, which is related to the ferroelectric–paraelectric phase transition. *ε*′ reached its maximum value (*ε*′ = 4400 for 1 kHz) at the Curie temperature and then decreased; however, the values of both parts of the complex dielectric permittivity still increased upon further increasing the temperature due to the presence of space-charge polarization in the material [38]. 

A slightly higher Curie temperature (300 K), than usually reported for pure SbSI crystals (283 K [6]–298 K [7]) was probably induced by stress between SbSI crystallites in the sample. A similar phenomenon has been previously observed for PbTiO_3_ nanotubes [39]. Another cause of the *T*_C_ shift may be the presence of air gaps or a non-crystallized phase between SbSI crystallites. The crystallization begins at the ampoule’s lateral surface and progresses radially to the center of the cylinder-shaped ampule along with the temperature gradient during the rapid cooling of the melted mass in liquid nitrogen; therefore, the crystallites overlapped and pressed each other in the center of the ampoule, and some impurities and empty spaces between crystallites may have formed during rapid cooling.

The calculated *ε*′ value of 4400 for 1 kHz is comparable to the value reported for SbSI thin films (4800) [40], and, as expected, is smaller than *ε*′ = 50,000 along the [001] direction obtained for SbSI single crystals [41]. Currently, no *ε*′ value has been reported for crystalline SbSI obtained by the rapid cooling of a melted mass. The expected value of *ε*′ for the examined sample should be higher than the one reported for a thin film and lower than the value reported for a SbSI single crystal. The smaller value of the dielectric constant real part than the SbSI thin film may be due to the imperfect alignment of SbSI crystallites in the sample. The *ε*′ value in SbSI is exactly three orders of magnitude higher along the [001] direction than along the [100] direction [8]. The sample was cut along the lateral plane parallel to the ampule ingot diameter. The crystallites were inaccurately reciprocally parallel in the sample due to their aforementioned radial distribution in the ampule.

### 3.2. Impedance Studies

**Figure 4:**
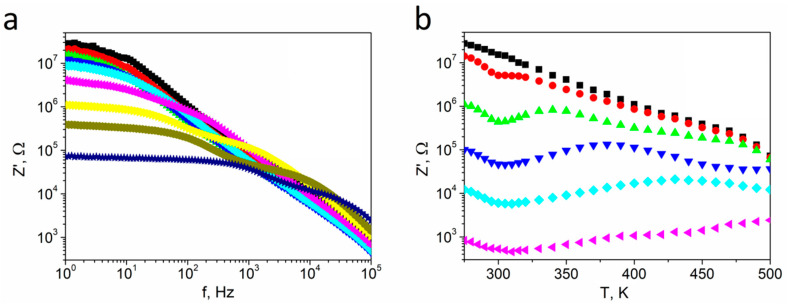
(**a**) Frequency spectra of various temperatures (■ 280 K; ⬤ 290 K; ▲ 300 K; **▼** 310 K; ♦ 320 K; ◀ 350 K; ► 400 K; ⬢ 450 K; ★ 500 K), and (**b**) temperature dependence for selected frequencies (■ 1 Hz; ⬤ 10 Hz; ▲ 100 Hz; **▼** 1 kHz; ♦ 10 kHz; ◀ 100 kHz;) of the real part of impedance for textured polycrystalline SbSI.

The frequency spectra of the real part of impedance (*Z*′) for various temperatures and the *Z*′ temperature dependence at selected frequencies of textured polycrystalline SbSI are presented in Figure 4.

Figure 4a shows the real part of impedance *Z*′ vs. frequency from 1 Hz to 100 kHz for selected temperatures over the range of 275–500 K. For low frequencies, a temperature-dependent *Z*′ plateau was observed. Then, the *Z*′ values monotonically decreased by approximately 1/*f* upon increasing frequency, which is typical for ferroelectric ceramics [42]. A step-like dependence of impedance appeared for temperatures higher than 350 K and moved towards higher frequencies as the temperature increased. In Figure 4b, *Z*′ monotonously decreased upon increasing the temperature at low frequencies (*f* = 1 Hz), which indicates the negative temperature coefficient of sample resistance, which is typical for semiconductors. Nevertheless, the temperature dependence of *Z*′ becomes more volatile as the frequency increased. The minimum value of *Z*′ was observed at 300 K for 10 Hz, which shifted to 310 K for 100 kHz. The following maximum value of *Z*′ displayed the same behavior but over a wider temperature range (shifting from 310 K for 10 Hz to 450 K for 10 kHz). In the case of measurements at 100 kHz, only an increase in the *Z*′ value was observed, and no maximum was visible over the entire examined temperature range. The relatively high values of *Z*′ at low frequencies at high temperatures are probably caused by a high defect concentration in the textured polycrystalline and charge accumulation at grain boundaries [43]. The significant decrease in *Z*′ values upon increasing the frequency (Figure 4a) confirms the presence of space-charge polarization [43], as indicated by the dielectric studies in Section 3.1. The space charges were depleted at high temperatures since they had already overcome the potential barrier in the regions of charge accumulation at grain boundaries, resulting in a peak [43,44].

**Figure 5:**
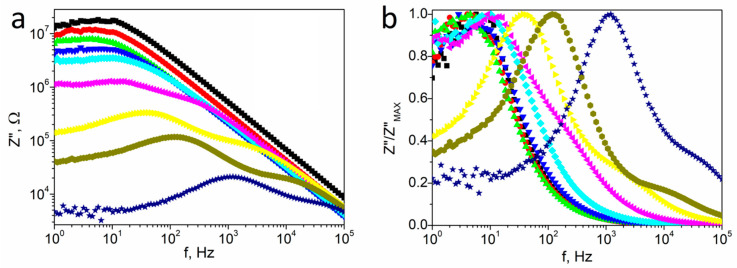
The frequency spectra of the imaginary part (*Z*″) of impedance (**a**), and normalized *Z*″/*Z*″_MAX_ function (**b**) at selected temperatures (■ 280 K; ⬤ 290 K; ▲ 300 K; **▼** 310 K; ♦ 320 K; ◀ 350 K; ► 400 K; ⬢ 450 K; ★ 500 K) for textured polycrystalline SbSI.

The frequency spectra of the imaginary part of impedance (*Z*″) at selected temperatures from 275 to 500 K (so-called loss-spectra) are shown in Figure 5a. The loss-spectra are congruent at low temperatures. The values of *Z*″ slightly increase as the frequency increases, reaching maximum values near 10 Hz before monotonically decreasing. The maximum moved towards higher frequencies at temperatures above 310 K. Simultaneously, its value decreased with increasing temperature due to a decrease in the resistance. Furthermore, the impedance value decreased by four orders of magnitude due to a thermal activation mechanism during this process. A strong broadening of the peak at higher frequencies was also visible above 330 K for the normalized *Z*″/*Z*″_MAX_ function (Figure 5b). The last peak broadened, as the temperature increased; however, a distinct maximum was not visible over the entire temperature range.

**Figure 6:**
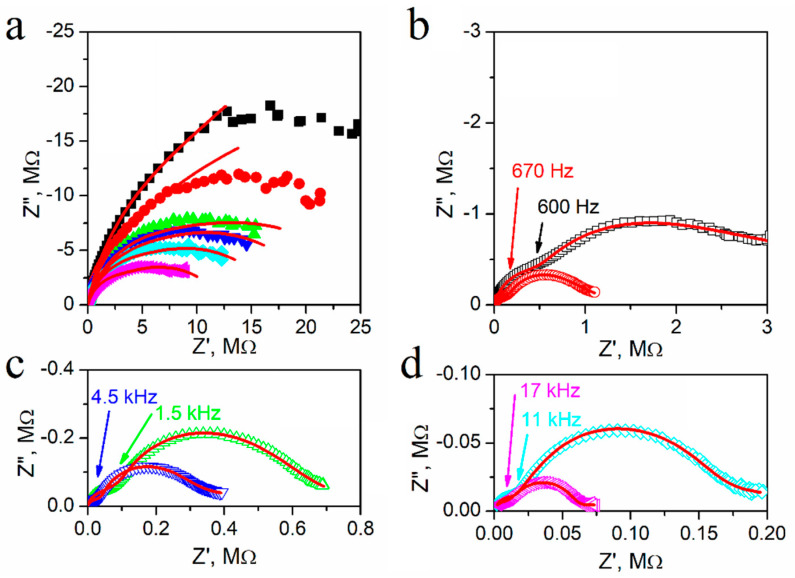
Nyquist plots of impedance (*Z*″ vs. *Z*′) for textured polycrystalline SbSI at selected temperatures (**a**) ■ 280 K; ⬤ 290 K; ▲ 300 K; **▼** 305 K; ♦ 310 K; ◀ 320 K; (**b**) ☐ 360 K; ◯ 400 K; (**c**) △ 420 K; ▽ 450 K; and (**d**) ◇ 480 K; ◁ 500 K. Red curves indicate the best fit of the experimental data to an equivalent circuit. Marked frequencies indicate the intersection of two semicircles arising for various frequency ranges (description in the text).

**Figure 7:**
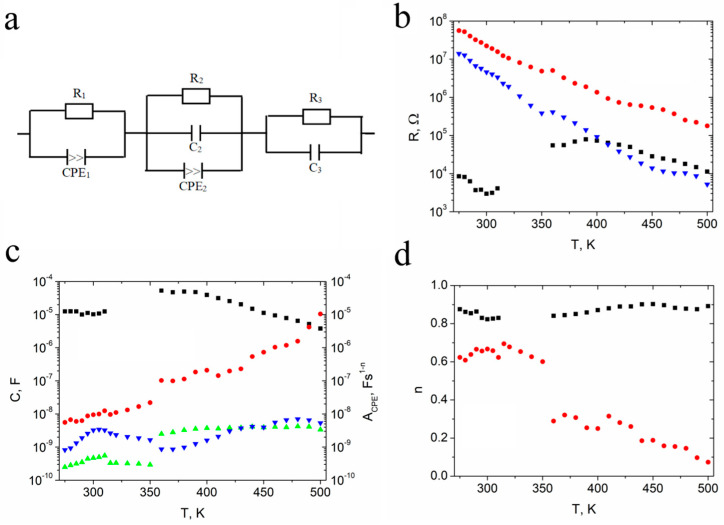
Equivalent circuit of textured polycrystalline SbSI (**a**) and temperature dependences of fitted parameters: resistance ■—R_1_; ⬤—R_2_; **▼**—R_3_; (**b**), capacitance ▲—C_2_; **▼**—C_3_; (left scale), A—constants of CPE element ■—A_CPE1_; ⬤—A_CPE2_; (right scale) (**c**), exponential indexes of CPE element ■—n_1_; ⬤—n_2_; (**d**) (description in the text).

Figure 6. presents the influence of temperature on the Nyquist plots of *Z*′ and *Z*″ for textured polycrystalline SbSI. An incomplete flattened semi-circle is visible at temperatures near *T*_c_ (Figure 6a). The Nyquist plot size decreases monotonically and appears to be composed of two overlapping semicircles as the temperature increased. Their separation becomes more apparent at higher temperatures (Figure 6b–d). The frequency values distinguishing between semicircles for different frequency ranges increased with the temperature, and these values have been indicated in Figure 6. Moreover, the size of the semicircle in the high-frequency region became smaller than the one at lower frequencies upon further increasing the temperature. A third semicircle appeared at low frequencies at higher temperatures (Figure 6c,d), which was associated with an electrode effect. The equivalent circuit was least-square fitted based on experimental data to distinguish effects arising in the sample. Figure 7 presents the proposed equivalent circuit and temperature dependences of the fitted parameters.

The equivalent circuit consists of three branches (Figure 7a). The first and second branches include the CPE element. The impedance of the constant phase element (CPE) is given by the equation:

(5) $$Z=\frac{1}{A{\left(i\omega \right)}^{n}}$$

where *i* is an imaginary unit, *ω* is the angular frequency, *A* is a constant, and *n* is an exponential index. 

The first parallel branch of the equivalent circuit (CPE_1_||R_1_) disappears in the temperature range from 315 K to 350 K. One can see the low resistance (Figure 7b) and high capacitance (Figure 7c) of this branch. The *n*-index value of the CPE_1_ element also indicates its capacitive character; therefore, for temperatures *T* < 315 K, this part of the circuit is responsible for the domain structure of the crystallites, while for *T* > 350 K, it may be attributed to electrode effects.

The resistance *R*_2_ is higher than resistance *R*_3_ (Figure 7b), and both significantly decreased upon increasing the temperature, as expected for a semiconducting material. The presence of the CPE_2_ element connected parallel with the capacitance *C*_2_ (Figure 7c) indicates that non-Debye relaxation behavior was observed. One can see, based on the *n*_2_ index of the CPE_2_ element that its character changed from capacitive into resistive upon increasing the temperature. The peak at the Curie temperature is visible on capacitance *C*_3_ (Figure 7c). The presence of two semicircular arcs associated with the electrical response due to grain interior and grain boundary is consistent with a brick-layer model for a polycrystalline material [45]. Considering the above, the second parallel branch of the equivalent circuit (CPE_2_||R_2_||C_2_) may be attributed to the conduction mechanism of grain boundaries, while the pure RC behavior of the third parallel branch (R_3_||C_3_) is associated with conduction inside crystallites.

The aforementioned multiple semicircular arcs were not clearly visible at low temperatures due to the high impedance of the sample, but they are evident in the electric modulus studies in the next section. 

### 3.3. Modulus studies 

Electric modulus formalism is an appropriate tool to study conductivity relaxation processes, which allows for the interpretation of bulk relaxation properties since changes in the large permittivity and conductivity values at low frequencies are negligible; therefore, electrode, space charge injection, and absorbed impurity conduction effects can even be ignored. The impedance analysis emphasizes the grain boundary conduction process in polycrystalline materials, while bulk effects are evident in the electric modulus frequency spectra [46]. The electric modulus formalism detects bulk properties as apparent conductivity relaxation times [47].

**Figure 8:**
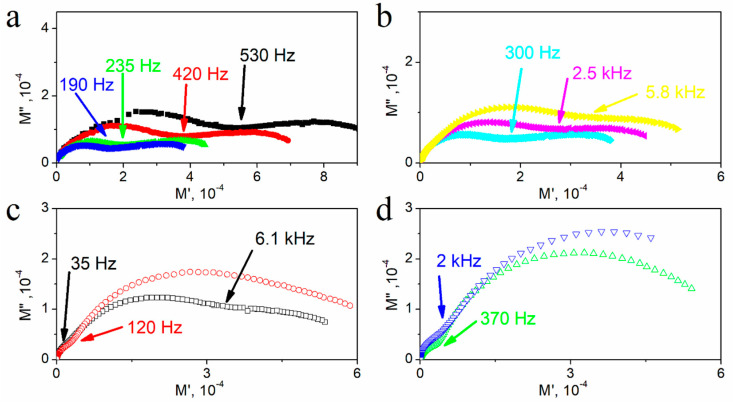
Complex electric modulus plots (*M*″ vs. *M*′) for textured polycrystalline SbSI at selected temperatures (**a**) ■ 275 K; ⬤ 285 K; ▲ 295 K; **▼** 305 K; (**b**) ♦ 310 K; ◀ 330 K; ► 350 K; (**c**) ☐ 360 K; ◯ 400 K; and (**d**) △ 450 K; ▽ 500 K. Marked frequencies indicate the intersection of semicircles arising for various frequency ranges (description in the text).

Figure 8 presents the complex electric modulus plots (*M*″ vs. *M*′) for textured polycrystalline SbSI at selected temperatures. Double, almost equal-sized semicircles are visible at 280 K (Figure 8a), in contrast to the single semicircle in the complex impedance plane at the same temperature (Figure 6a). This indicates the presence of two relaxation effects of comparable capacity but different resistance. The frequency value at the intersection of these semicircles has been indicated in Figure 8 at every temperature. In the electric modulus plots, the higher-frequency semicircle is assigned to the grains, while the lower-frequency one is assigned to the grain boundaries [48]. Depressed arcs indicate the distribution of relaxation times. At first, the size of semicircles decreased as the temperature increased (Figure 8a), reaching the minimum before beginning to increase (Figure 8b–d). The higher-frequency semicircle starts to increase above 310 K and the lower-frequency one above 320 K, and another low-frequency semicircle starts to appear at 350 K. The presence of two semicircles intersections is visible and indicated in Figure 8c at 360 K. At temperatures above 400 K, the higher-frequency semicircle disappears, and the low-frequency one becomes more pronounced.

**Figure 9:**
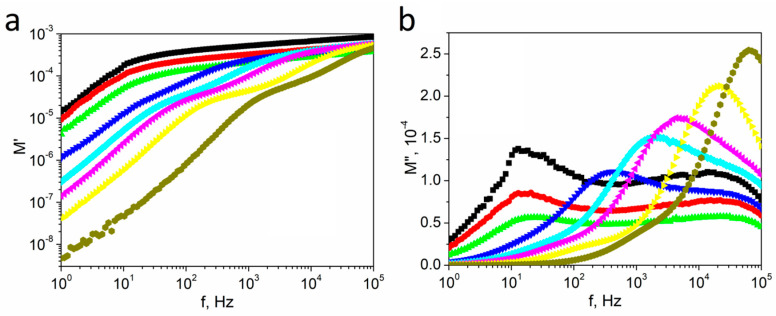
The frequency spectra of the real *M*′ (**a**) and imaginary *M*″ (**b**) parts of electrical modulus at selected temperatures (■ 280 K; ⬤ 290 K; ▲ 310 K; **▼** 350 K; ♦ 380 K; ◀ 400 K; ► 450 K; ⬢ 500 K) for textured polycrystalline SbSI.

The frequency spectra of the real *M*′ and imaginary *M*″ parts of the electrical modulus at different temperatures for textured polycrystalline SbSI are presented in Figure 9. The value of *M*′ increased monotonously, indicating continuous dispersion as the frequency increased until reaching a nearly constant value for high frequencies at all temperatures. This shape of the spectrum may be assigned to conduction phenomena due to the short-range mobility of charge carriers [49]. 

Two separate maximum values of *M*″(*f*) plots are visible for ~10 Hz and ~10 kHz at low temperatures, and *M*″ tends to be zero at low frequencies, which indicates that electrode polarization is negligible or absent [50]. The low-frequency peak position was fixed upon increasing the temperature to the Curie temperature, and then it started to move towards higher frequencies. The position of the high-frequency peak was independent of temperature, but it disappeared at high temperatures because it overlapped with the moving low-frequency peak. Simultaneously, a further extension of the peak was observed at the low-frequency side. 

**Figure 10:**
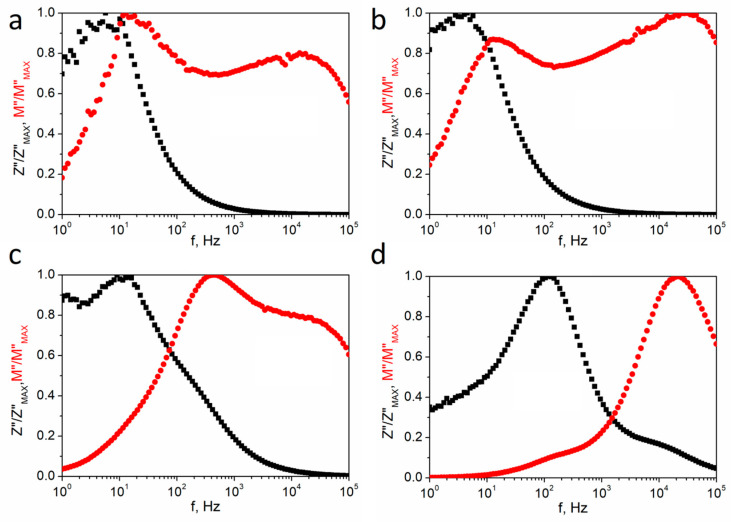
Normalized imaginary parts of the impedance *Z*″/*Z*″_MAX_ (■) and the electric modulus *M*″/*M*″_MAX_ (⬤) spectra for textured polycrystalline SbSI at 280 K (**a**), 300 K (**b**), 350 K (**c**), and 450 K (**d**).

A comparison of normalized imaginary parts of the impedance *Z*″/*Z*″_MAX_ and normalized imaginary parts of the electric modulus *M*″/*M*″_MAX_ as a function of frequency at the selected temperatures for textured polycrystalline SbSI is presented in Figure 10. These plots confirm that non-Debye relaxation behavior was observed because the *Z*″ and *M*″ curves do not coincide when plotted as a function of frequency at a single temperature. The maximum of *M″* appears at a much higher frequency than for *Z*″. The magnitude of mismatch of the *M*″ and *Z*″ parameters represents a change in the apparent polarization. The difference in the peak position of the normalized parameters in the frequency domain indicates the conductive path [51]. The peaks of the *M*″ and *Z*″ spectra appear at the same frequency for the long-range process, but at various frequencies in the case of the localized process [45].

**Figure 11:**
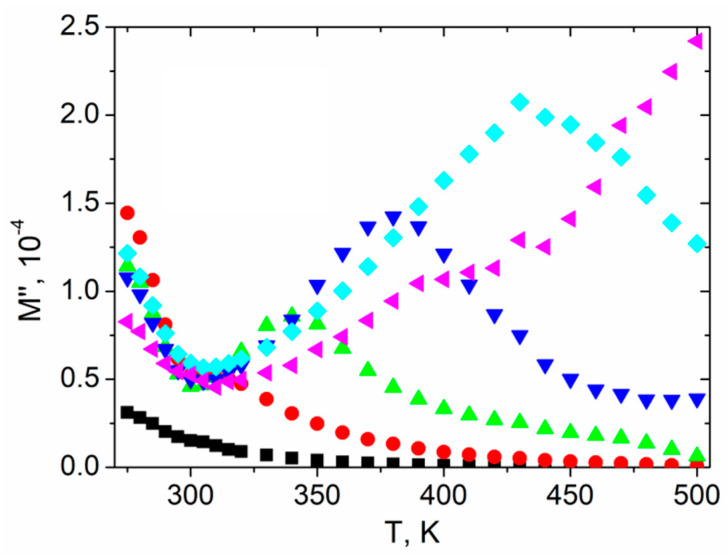
The imaginary part of electrical modulus *M*″ vs. temperature of textured polycrystalline SbSI for the selected frequencies (■ 1 Hz; ⬤ 10 Hz; ▲ 100 Hz; **▼** 1 kHz; ♦ 10 kHz; ◀ 100 kHz).

Figure 11 presents the temperature dependence of the imaginary *M*″ part of the electrical modulus for chosen frequencies. Each characteristic reached its minimum value at the Curie temperature and then a peak appeared for higher temperatures, except the plots for 1 Hz and 100 kHz, where a minimum and peak were not present, respectively. The peak shifted toward a higher temperature as the frequency increased, which was attributed to the conduction process [52].

### 3.4. ac Conductivity Studies

The frequency spectra of the ac conductivity provide information about the nature of charge carriers [53]. Jonscher attempted to explain the behavior of ac conductivity using the following law [53]:

(6) $$\sigma_{ac}\left(\omega \right)=\sigma \left(0\right)+\sigma_{1}\left(\omega \right)=\sigma_{dc}+a\omega^{n}$$

where *σ_ac_*(*ω*) is the total measured conductivity; *σ*(0), σ_dc_ is a frequency-independent term giving dc conductivity, and *σ*_1_(*ω*) is the pure dispersive component of ac conductivity having a power-law characteristic in the angular frequency ω domain with an exponent *n*, and *a* is a proportionality factor. The value of *n* is in the range of 0 < *n* <1 and is frequency-independent but temperature- and material-dependent [53].

**Figure 12:**
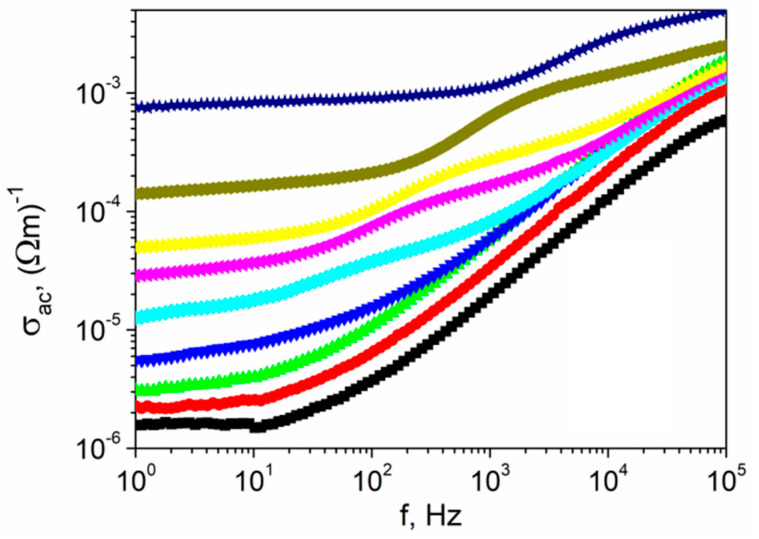
The frequency spectra of the total measured conductivity for textured polycrystalline SbSI at selected temperatures (■ 280 K; ⬤ 290 K; ▲ 300 K; **▼** 320 K; ♦ 350 K; ◀ 380 K; ► 400 K; ⬢ 450 K; ★ 500 K).

The ac conductivity vs. frequency plots (so-called conductivity spectra) for textured polycrystalline SbSI at various temperatures presented in Figure 12 show that dispersion is evident in the conductivity spectra at each temperature. Moreover, their shape changes at 320 K. Below this temperature, *σ*_ac_ decreased upon decreasing the frequency and becomes nearly independent for low frequencies at temperatures lower than 320 K. The phenomenon of increasing conductivity with increasing frequency (Figure 12) may be interpreted in terms of the Schottky barrier at the metal–dielectric interface, Maxwell–Wagner type conduction, and hopping conduction [54]. The positive slope of ac conductivity with frequency is also attributed to the cation disorder between neighboring sites, i.e., neighboring grains and their boundaries, in a sample and the presence of space charges at these temperatures. The conduction dependence obeys the Jonscher universal power law (Equation (6)), with *n* ≈ 0.8. According to Jonscher’s law [53], the frequency-dependent conductivity originates from relaxation phenomena due to the mobility of charge carriers. When a mobile charge hops to a new site from its initial position, it remains in a displaced state between two potential energy minima. The value of *n* < 1 means that hopping involves a translational motion with a sudden hopping [53]. The value of *n* = 0.8 is observed if a material contains dipoles that can point in two or more directions [48], which may be caused by one or more of the following in the case of SbSI polycrystals:

- Alternating arrangement of polar double chains [(SbSI)_∞_]_2_ parallel to the [001] axis above the Curie point inside a single crystallite [15];
- Atoms shifting from the mirror plane perpendicularly to the [001] direction in the paraelectric phase inside a single crystallite [16];
- Heterogeneous [Sb(S,I)]_∞_ chains in the crystal structure consisting of sections with different lengths and opposite polarity resulting in positively or negatively-polarized irregular nanodomains inside a single crystallite [16], which are also visible in polarized light transmission [55]. Localized electron states may be created at ferroelectric domain boundaries. Domain walls create defects in the crystal lattice, which results in the appearance of new local levels inside the energy gap and potential barriers inside the crystal. It is visible as an electrical conductivity change when electrons may localize on domain boundaries at optical transitions to and inside the band [56];
- Coexistence of paraelectric and ferroelectric phases inside a single crystalline in the region of ferroelectric phase transition within a temperature range of several degrees [57]. The temperature range of both phases coexistence increases linearly upon increasing the charge concentration [58];
- The aforementioned inaccurately parallel alignment of crystallites in the ampule.

A slope change is visible at temperatures above 320 K, and it moves towards a higher frequency as the temperature increases (Figure 12). This frequency is known as the hopping frequency of the polarons (*ω_p_*) and is temperature-dependent [53].

**Figure 13:**
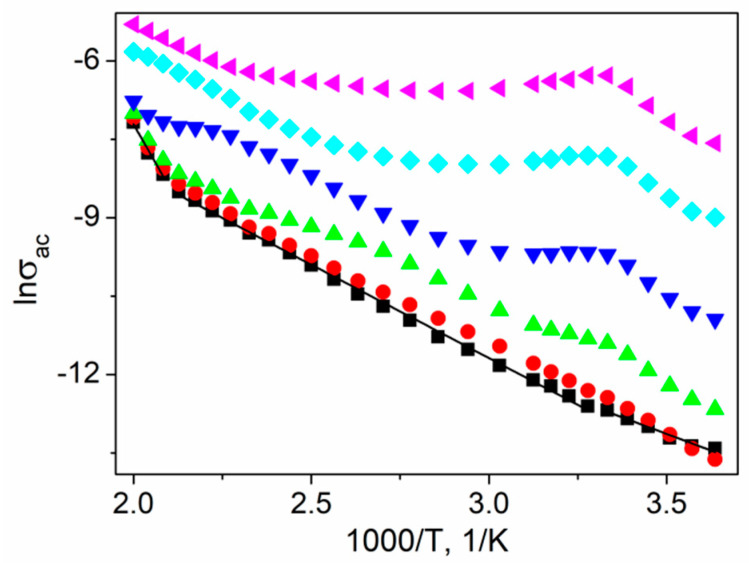
The Arrhenius plot of σ_ac_ for a textured polycrystalline SbSI for various frequencies (■ 1 Hz; ⬤ 10 Hz; ▲ 100 Hz; **▼** 1 kHz; ♦ 10 kHz; ◀100 kHz;). Straight lines (▬) represent the least-squares fitted theoretical dependencies for separate temperature ranges (description in the text).

Figure 13 presents the selected ln(*σ*_ac_) vs. reciprocal temperature for certain frequencies. For low frequencies (~1 Hz) the dependency is linear, but one can distinguish three regions characterized by different slopes (I: *T* < 300 K; II: 305 < *T* < 460 K; III: *T* > 480 K). Considering the relationship:

(7) $$\sigma_{\mathrm{ac}}=\sigma_{0}\exp(-\frac{E_{a}}{k_{B}T})$$

where *E*_a_ is the activation energy of conductivity, σ_0_ is a pre-exponential factor, and *k*_B_ is the Boltzmann constant, the obtained values of activation energy are estimated as *E*_a(I)_ = 0.192(41) eV, *E*_a(II)_ = 0.308(35) eV, and *E*_a(III)_ = 1.03(12) eV in each temperature range, respectively. The phase transition anomaly appears, and the ac conductivity attained a local maximum at 300 K as the frequency increased (Figure 13). 

### 3.5. Relaxational Analysis

The peaks in the frequency-dependent *Z*″ (Figure 5a) and *M*″ spectra (Figure 9b) indicate relaxation phenomena in the system. One can determine the most probable relaxation time (*τ_Z_*) and (*τ_Μ_*) from the loss peak position in the *Z*″ and the *M*″ spectra, respectively, by using the relation [28]:

(8) $$2\pi f_{\mathrm{MAX}}\tau =1$$

where *f*_MAX_ is the relaxation frequency indicating the peak position.

**Figure 14:**
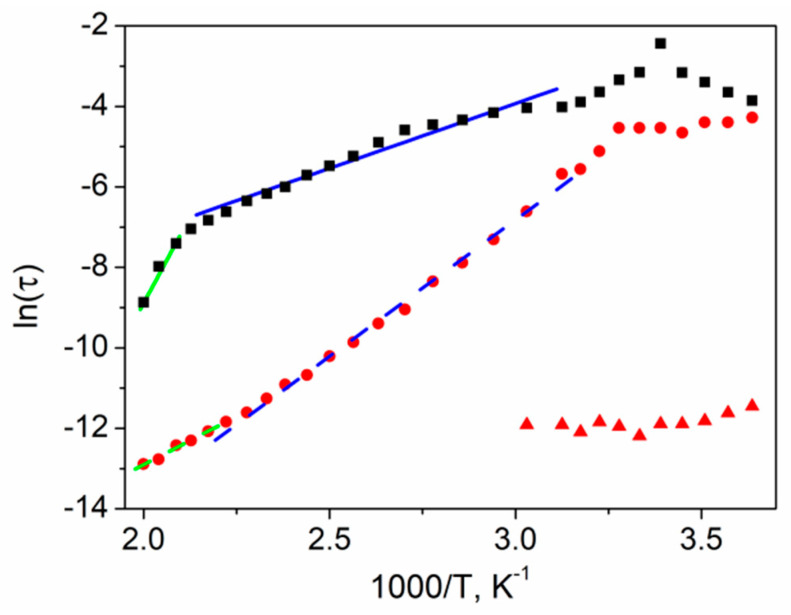
The Arrhenius plot of relaxation time *τ_Z_* (■) and *τ_Μ_* for the entire temperature range (⬤) and another for *T* < 330 K (▲) for textured polycrystalline SbSI. Blue (▬) and green (▬) straight, solid lines represent the least-squares fitted theoretical *τ_Z_* dependencies at 330 K < *T* < 450 K and 460 K < *T* < 500 K, respectively. Blue (--) and green (--) dashed, straight lines represent the least-squares fitted theoretical *τ_Μ_* dependencies at 330 K < *T* < 450 K and 460 K < *T* < 500 K, respectively (description in the text).

The temperature dependence of *τ_Z_* and *τ_Μ_* relaxation times of textured polycrystalline SbSI is presented in Figure 14. A singular relaxation time can be specified in the case of the *Z*″ spectrum, while in the case of the *M*″ spectrum, two separate relaxation times can be distinguished at *T* < 330 K. The reciprocal temperature plots of calculated relaxation times are presented in Figure 14.

The relaxation time *τ_Z_* reached the maximum value at the Curie temperature. The straight lines in Figure 14 present the least-squares fitted Arrhenius dependencies *τ* within each of the selected linear temperature ranges for textured polycrystalline SbSI:

(9) $$\tau_{}=\tau_{0}\exp(\frac{E_{a}}{k_{B}T})$$

where $\tau_{0}$ is a pre-exponential factor, *E*_a_ is the activation energy of conductivity, and *k*_B_ is the Boltzmann constant. The calculated activation energies are presented in Table 1.

Activation energies estimated based on *τ_Z_* are in good agreement with the values calculated from the *σ_ac_* Arrhenius plot (Figure 13). The sample electrical conductivity changed from frequency-independent dc conductivity to frequency-dependent ac conductivity as the frequency increased; therefore, the structure of the material, its dielectric properties, and the charge carrier's behavior under an ac field play a significant role. The magnitude of thermal fluctuation of the crystal lattice increases with the temperature, resulting in a higher probability of electric charge-phonon interactions and, consequently, a mobility decrease. On the other hand, increasing the temperature resulted in a higher probability of carriers escaping from potential wells and become mobile starting from the outer bands, resulting in a conductivity increase typical of semiconductors (Figure 13). Simultaneously, at higher temperatures, there were fewer charges available in the outer bands, resulting in increased activation energy, especially in semiconductors characterized by low conductivities like SbSI; therefore, the temperature greatly affected the ac conductivity, as well as the activation energy of the material. These phonon-assisted hopping-type conduction mechanisms are dominant here in the case of impedance phenomena related to *τ_Z_* values. The charge carriers hop between the traps situated in the bandgap of the material, and *τ_Z_* is the effective relaxation time [54].

The *τ_Μ_* values are related to polarization phenomena, dielectric permittivity, and complex electric modulus measurements. The high *ε* values indicate that space charges produced only a relatively weak electric field. Similarly, charged scattering centers played a secondary role because they created a relatively weak field at high *ε*. In contrast, neutral scattering centers are important. In the case of conduction electron scattering on hydrogen-like impurities, the diameter and polarizability increased, while the activation energy decreased upon increasing *ε*, so that inelastic charge scattering is also possible [59]. *ε* increased in an examined sample (Figure 3), while the activation energy calculated from *τ_Μ_* Arrhenius dependence decreased (Table 1) upon increasing the temperature. This scattering mechanism is proportional to the electric field strength where electron-phonon coupling occurs (red triangles in Figure 14). At the same time, the scattering of electric charges on low-energy transverse optical phonons polarized perpendicularly to the double SbSI chains may occur [59], which is dominant at high temperatures (red circles in Figure 14); therefore *τ_Μ_* values can be associated with an effective relaxation time of this mechanism. This frequency is several orders of magnitude higher than the one calculated from the expected *τ_Z_* dependence.

## 4. Conclusions

In this article, for the first time, impedance and electric modulus formalism has been applied to describe phenomena in SbSI polycrystals. Complete studies of dielectric permittivity, conductivity, and relaxational analysis of this material using complex impedance spectroscopy were presented for the first time. These measurements were performed along the [001] crystallographic direction, i.e., along the *c*-axis of polycrystals, where they exhibit the highest values of tensor components of many interesting physical properties, and along which is the most frequently applied in devices, e.g., piezoelectric transducers. A comprehensive analysis and explanation of the results were presented. The only phase transition was observed at 300 K. The interaction and overlapping of crystallites resulting in a stress-induced Curie temperature shift, as well as the possible incidence of an air gap between crystallites, were pointed out as reasons for the small *T*_C_ shift. The influence of the polycrystal growth process on the obtained characteristics and calculated *ε* values was discussed. The hopping conduction mechanism was interpreted considering the crystal internal structure, coexistence of paraelectric and ferroelectric phases, and interactions between crystallites in a sample. The charge carriers hopping in ferroelectrics was associated not only with hopping centers as with common semiconductors, but it may occur between grains and their boundaries, ferroelectric domains and their boundaries, as well as coexisting para- and ferroelectric regions and their boundaries, depending on the sample temperature. Additional effects should be considered in the case of other parameters affecting charge concentration or mobility, e.g., the influence of external pressure or the Barkhausen effect in the case of illuminated samples in the ferroelectric phase.

## Figures and Tables

**Table 1:** The activation energy of conductivities at various temperatures estimated from *τ_Z_* and *τ_Μ_* for textured polycrystalline SbSI.

Relaxation Time	*E*_a_ for 330 K < *T* < 450 K	*E*_a_ for 460 K < *T* < 500 K
*τ_Z_*	0.277(14) eV	1.48(13) eV
*τ_Μ_*	0.584(52) eV	0.414(21) eV

## Data Availability

Raw data were generated at Silesian University of Technology, Institute of Physics. The data presented in this study are available on request from the corresponding author.
